# Optical coherence tomography (OCT) - versus angiography-guided strategy for percutaneous coronary intervention: a meta-analysis of randomized trials

**DOI:** 10.1186/s12872-024-03930-y

**Published:** 2024-05-20

**Authors:** Yanwei Wang, Xi Yang, Yutao Wu, Yanqin Li, Yijiang Zhou

**Affiliations:** 1 Department of Cardiology, Ningbo Medical Treatment Center Lihuili Hospital, 57 Xingning Road, Ningbo, 315000 PR China; 2 Department of Cardiology, The First Affiliated Hospital, School of Medicine, Zhejiang University, 79 Qingchun Road, Hangzhou, 310003 Zhejiang PR China; 3 Department of Coronary Care Unit, Ningbo Medical Treatment Center Lihuili Hospital, 57 Xingning Road, Ningbo, 315000 PR China

**Keywords:** Optical coherence tomography, Coronary angiography, Coronary artery disease, Percutaneous coronary intervention

## Abstract

**Background:**

Optical coherence tomography (OCT) guidance in percutaneous coronary intervention (PCI) has been shown to improve procedural outcomes. However, evidence supporting its superiority over angiography-guided PCI in terms of clinical outcomes is still emerging and limited. This study aimed to compare the efficacy and safety of OCT-guided PCI versus angiography‐guided PCI in patients with coronary artery disease (CAD).

**Methods:**

A systematic search of electronic databases was conducted to identify randomized control trials (RCTs) comparing the clinical outcomes of OCT-guided and angiography‐guided PCI in patients with CAD. Clinical endpoints including all-cause mortality, myocardial infarction (MI), target lesion revascularization (TLR), stent thrombosis and major adverse cardiac events (MACE) were assessed.

**Results:**

Eleven RCTs, comprising 2,699 patients in the OCT-guided group and 2,968 patients in the angiography-guided group met inclusion criteria. OCT-guided PCI was associated with significantly lower rates of cardiovascular death(RR 0.56; 95%CI: 0.32–0.98; *p* = 0.04; I^2^ = 0%), stent thrombosis(RR 0.56; 95%CI: 0.33–0.95; *p* = 0.03; I^2^ = 0%), and MACE (RR 0.79; 95%CI: 0.66–0.95; *p* = 0.01; I^2^ = 5%). The incidence of all-cause death (RR 0.71; 95%CI: 0.49–1.02; *p* = 0.06; I^2^ = 0%), myocardial infarction (RR 0.86; 95%CI: 0.67–1.10; *p* = 0.22; I^2^ = 0%) and TLR (RR 0.98; 95%CI: 0.73–1.33; *p* = 0.91; I^2^ = 0%) was non-significantly lower in the OCT-guided group.

**Conclusions:**

Among patients undergoing PCI, OCT-guided PCI was associated with lower incidences of cardiovascular death, stent thrombosis and MACE compared to angiography-guided PCI.

**Trial registration:**

PROSPERO registration number: CRD42023484342.

**Supplementary Information:**

The online version contains supplementary material available at 10.1186/s12872-024-03930-y.

## Introduction

Percutaneous coronary intervention (PCI) using second-generation drug-eluting stents (DES) under angiographic guidance has significantly reduced the rates of stent-related or target-vessel adverse clinical events [1]. Unlike angiography, intravascular imaging techniques such as optical coherence tomography (OCT) and intravascular ultrasound (IVUS), offer unique advantages in assessing lesion characteristics, enhancing procedural optimization [2]. Recent studies have indicated that PCI under guidance of intravascular imaging may lead to improved cardiovascular outcomes, including reduced in-hospital mortality, myocardial infarction, and target-lesion revascularization [3, 4].

Optical coherence tomography is a light-based modality of intravascular imaging with superior axial resolution compared to IVUS (15 vs. 150 mm), enabling detailed assessment of plaque components and precise measurements of vessel and stent dimensions [5]. OCT’s accurate surpasses that of angiography and IVUS in identifying subtle morphological details such as malapposition, residual thrombus, plaque prolapse, and residual dissections, contributing to improved procedural outcomes [6]. In instances of stent failure, OCT should be considered to identify and address underlying mechanical factors [7]. However, evidence supporting OCT guidance in PCI is promising yet limited albeit. Buccheri et al. found a significant reduction of major adverse cardiac events and cardiac death with OCT compared to coronary angiography (CA) in a meta-analysis of 14 observational studies, although a sensitivity analysis restricted to RCTs showed no significant difference in outcomes [2]. Park et al.‘s network meta-analysis, comparing IVUS-, OCT-, and CA-guided PCI, revealed OCT and IVUS to be comparable across all endpoints, with OCT’s superiority over CA unconfirmed due to the small size and limited number of studies involving OCT, affecting the power to detect its potential benefits [8].

The recent publication of two well-designed randomized control trials (RCTs) ILUMIEN IV OPTIMAL PCI and OCTOBER [9, 10], has contributed additional evidence on the efficacy of OCT-guided coronary intervention. With updated clinical data, we conducted a meta-analysis to directly compare OCT‐guided versus angiography‐guided PCI.

## Methods

This meta-analysis was conducted in accordance with the Preferred Reporting Items for Systematic Reviews and Meta-Analyses (PRISMA) checklist [11]. Systematic database search was performed on PubMed, Web of Science and ClinicalTrials.gov for relevant articles using keywords such as: “Optical Coherence Tomography”, “OCT”, “Intravascular Imaging”, “PCI”, “Percutaneous Coronary Intervention” and “Angiography”. Additionally, references from pertinent studies, reviews, editorials, letters, and related conference abstracts were also reviewed.

Inclusion criteria were RCTs directly comparing clinical outcomes between OCT-guided and angiography-guided PCI, encompassing all clinical presentations. Studies not in English or involving non-human subjects or lacking clinical data were excluded. Titles and abstracts were initially screened; full text articles were reviewed if the study appeared potentially eligible.

Efficacy endpoints included all-cause mortality, cardiovascular death, myocardial infarction (MI), target lesion revascularization (TLR), stent thrombosis, and major adverse cardiac events (MACE), each defined as per the original study.

Two investigators independently assessed reports for eligibility at title and/or at abstract level, with divergences resolved by a third reviewer. The risk of bias was evaluated by the same two reviewers, in accordance with the Cochrane Collaboration methods [12].

Data analysis was performed with Review Manager (Rev-Man, version 5.3, the Nordic Cochrane Centre, The Cochrane Collaboration, 2013). We calculated risk ratios (RR) with 95% confidence intervals (CI) from reported event frequencies. Heterogeneity among trial results was assessed using the Chi [2] statistic for heterogeneity and the I [2] index for inconsistency. Results were reported as the *p*-value from the Chi [2] test (*p* < 0.05 indicating significant heterogeneity) and the I [2] value, with interpretations of low (0–25%), moderate (50–75%), and high (> 75%) inconsistency levels. In the absence of significant heterogeneity (*P* > 0.1 and I^2^ < 50%), RR and CI were combined using a fixed-effect model. If significant heterogeneity was present (*P* ≤ 0.1 and I^2^ ≥ 50%), the random-effects model (Mantel-Haenszel) was used. To explore potential causes of study heterogeneity, subgroup analysis focusing on ACS patients and those receiving new-generation DES implantation was performed. Additionally, a sensitivity analysis was conducted to verify that studies with a serious risk of bias did not influence the main analysis outcomes. To evaluate potential publication bias, funnel plots were generated to reflect the trial results against their precision.

## Results

**Fig. 1:**
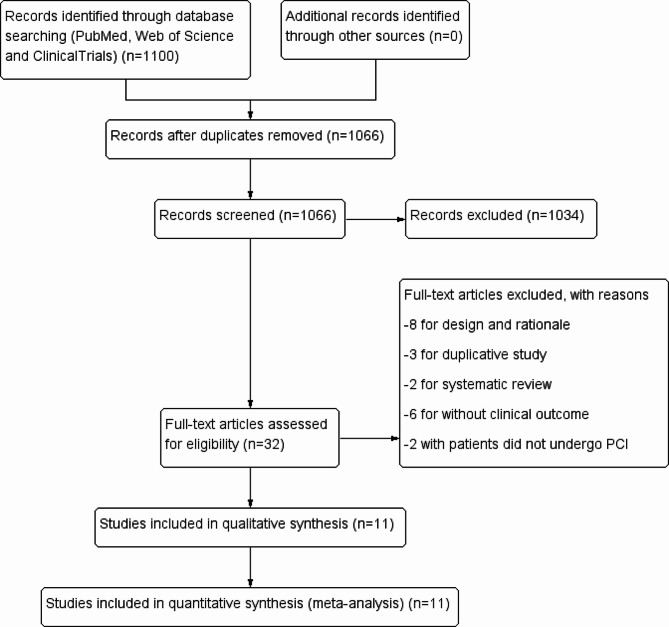
Flowchart of study selection

Following deduplication, titles and abstracts screening, and full-text review based on predefined inclusion and exclusion criteria, 11 RCT studies [9, 10, 13–21] involving 5,667 patients were qualified for analysis (Fig. 1). The detailed characteristics of the included studies are shown in Table 1. Studies varied with regard to the year of publication, clinical presentation, type of stents, and duration of follow up. In general, 2,699 (47.6%) patients underwent OCT-guided PCI, while 2,968 (52.4%) received angiography-guided PCI. Four studies [14, 17, 18, 21] exclusively focused on patients with acute coronary syndrome (ACS), while two studies [9, 13] primarily addressed complex coronary-artery lesions. Nine trials exclusively used drug eluting stents, whereas two trials [14, 16] employed only biodegradable stents.

**Table 1:** The characteristics of the study included

Trial	ILUMIEN IV [10]	OCTOBER [9]	RENOVATE-COMPLEX-PCI [13]	HONEST [14]	iSIGHT [15]	OPTICO BVS [16]
Year	2023	2023	2023	2022	2021	2020
n (OCT/Angiography)	1233/1254	600/601	278/547	37/38	51/49	19/19
Type of stent	DES	DES	BVS/DES	MBRS	DES	BVS
Clinical situation	High-risk patient	Complex bifurcation lesion	Complex coronary-artery lesions	STEMI	PCI of native coronary arteries	Coronary artery disease
Follow up (months) (mean)	24 (729 days)	24 (2 years)	24 (2.1 years)	6 (6 months)	24 (2.5 years)	12 (1 year)
***Clinical Baseline*** (OCT/Angiography)						
Age (years)	65.5 ± 10.5/65.7 ± 10.3	66.4 ± 10.5/66.2 ± 9.9	/66.0 ± 10.0	61.1 ± 10.9/61.7 ± 10.1	59.9 ± 8.9/58.6 ± 10.2	63.3 ± 12.7/62.9 ± 9.1
Male (%)	78.5/76.2	78.8/79.0	NA/78.8	78.4/81.6	60.8/77.5	79/79
Diabetes (%)	42.4/41.5	17.2/16.1	NA/40.8	5.4/10.5	33.3/44.9	21/21
Hyperlipidemia (%)	65.5/68.6	NA	NA/51.2	40.5/34.2	70.6/57.2	68/63
Hypertension (%)	71.4/74	72.4/70.3	NA/59	40.5/42.1	90.2/79.6	37/58
Dialysis (%)	2.2/1.9	2.0/2.3	NA	NA	0/0	NA
Current smoker (%)	19.6/19.7	12.8/14.1	NA/17.4	27/31.6	33.3/28.6	37/32
Prior PCI (%)	13.3/13.4	40.7/42.8	NA/23.2	10.8/10.5	NA	32/16
***Lesion characteristics*** (OCT/Angiography)						
Severe calcification (%)	11.4/11.7	NA	NA/14.3	0/0	NA	0/0
Two-stent bifurcation (%)	3.2/3.4	65/63.6	NA	0/0	0/0	0/0
***Procedural Data*** (OCT/Angiography)						
Total stent length (mm)	44.2 ± 23.8/40.5 ± 24.0	38(28,51)/33(23,48)	NA	NA	28.6 ± 12.0/25.8 ± 10.4	NA
Procedure time (min)	68.3 ± 38.3/50.0 ± 35.4	NA	70(51,95)/53.5(40,75)	58(48,76)/41(32,60)	58.6 ± 17.3/50.7 ± 21.1	72(56,94)/40(35,54)
Contrast volume used (ml)	231.9 ± 88.2/198.3 ± 81.7	NA	214.2 ± 118.5/193.7 ± 113.3	190.7 ± 80.6/139.1 ± 71.1	94.1 ± 40.5/72.3 ± 35.8	284(267,355)/211(191,300)

Trial	OCT STEMI (ROBUST) [17]	DOCTORS [18]	ILUMIEN III: OPTIMIZE PCI [19]	OCTACS [21]	Kim [20]
Year	2018	2016	2016	2015	2014
n (OCT/Angiography)	105/96	120/120	158/140	40/45	58/59
Type of stent	DES	BMS/DES	DES	DES	DES
Clinical situation	STEMI	NSTEMI	PCI of native coronary arteries	NSTEMI	Coronary artery disease
Follow up (months) (mean)	9 (9 months)	6 (6 months)	1 (30 days)	6 (187 days)	12 (12 months)
***Clinical Baseline*** (OCT/Angiography)					
Age (years)	57(46,70)/59(47,72)	60.8 ± 11.5/60.2 ± 11.3	66(59,72)/67(56,74)	61.8 ± 9.4/62.6 ± 11.0	58.8 ± 10.8/61.6 ± 9.7
Male (%)	83/87	75.8/77.5	69/73	72/68	78/72.5
Diabetes (%)	17/26	21.7/15.8	33/29	16/10	32/31.4
Hyperlipidemia (%)	NA	49.2/46.7	73/77	44/38	66/72.5
Hypertension (%)	50/52	55.8/41.7	78/75	56/56	54/49
Dialysis (%)	NA	NA	8/8	NA	0/0
Current smoker (%)	64/59	39.2/42.5	18/24	46/36	32/29.4
Prior PCI (%)	4/4	NA	7/10	6/4	NA
***Lesion characteristics*** (OCT/Angiography)					
Severe calcification (%)	NA	0/0	20/26	0/0	0/0
Two-stent bifurcation (%)	NA	0/0	0/0	0/0	0/0
***Procedural Data*** (OCT/Angiography)					
Total stent length (mm)	NA	17.9 ± 5.6/17.3 ± 5.5	23.5(15,32)/20(16,30)	22.6 ± 9.0/20.1 ± 8.4	18.0 ± 3.9/17.6 ± 4.3
Procedure time (min)	NA	56(49,77)/36(25,50)	71(57,101)/57.5(39,78)	44(29.5,57.8)/31(29.8,39.3)	NA
Contrast volume used (ml)	230(193,270)/ 168(130,190)	190(140,250)/ 120(90,160)	222(164,285)/ 183(140,250)	150(100,255)/ 110(100,152.5)	NA

BVS = Bioresorbable vascular scaffold; BMS = Bare metal stent; DES = Drug-eluting stents; MBRS = Magnesium bioresorbable scaffold; NSTEMI = Non-ST-segment elevation myocardial infarction; PCI = Percutaneous coronary intervention; STEMI = ST-segment elevation myocardial infarction

**Fig. 2:**
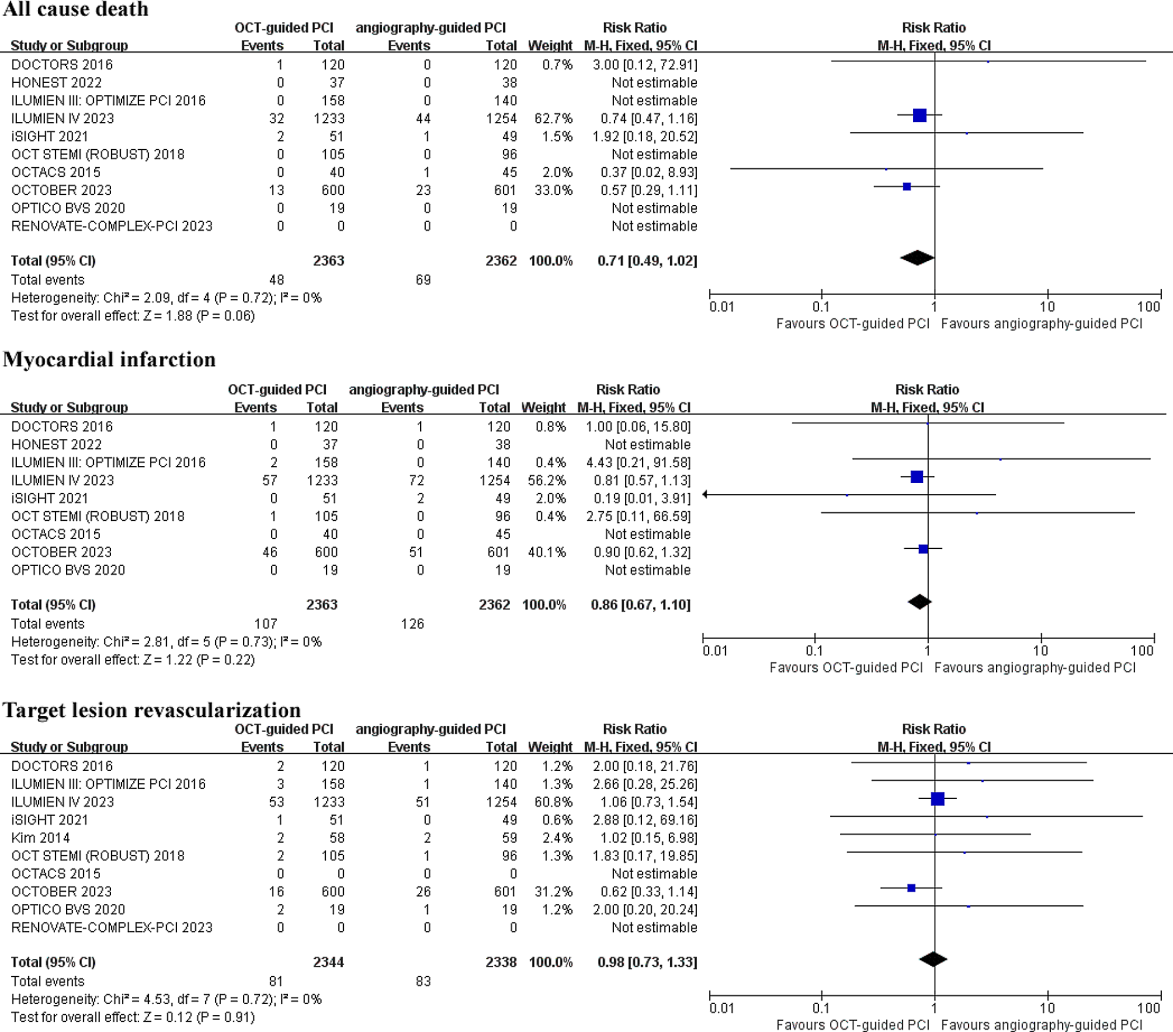
Forest plot for incidence of all cause death, myocardial infarction (MI) and target lesion revascularization (TLR). Risk ratio for individual studies (squares) and meta-analysis (diamonds) and 95% CI (horizontal lines) are presented

For all-cause mortality, ten studies involving 4,725 patients found that OCT-guided PCI non-significantly reduced all-cause mortality compared to CA guidance, with a relative risk (RR) of 0.71 (RR 0.71; 95% CI: 0.49–1.02; *p* = 0.06; I^2^ = 0%) (Fig. 2).

Similarly, for myocardial infarction, the same ten studies showed no significant difference in incidence between OCT-guided and CA-guided PCI (RR 0.86; 95% CI: 0.67–1.10; *p* = 0.22; I^2^ = 0%) (Fig. 2).

Another ten studies involving 4,682 patients also reported no significant difference in target lesion revascularization (RR 0.98; 95% CI: 0.73–1.33; *p* = 0.91; I^2^ = 0%) (Fig. 2).

**Fig. 3:**
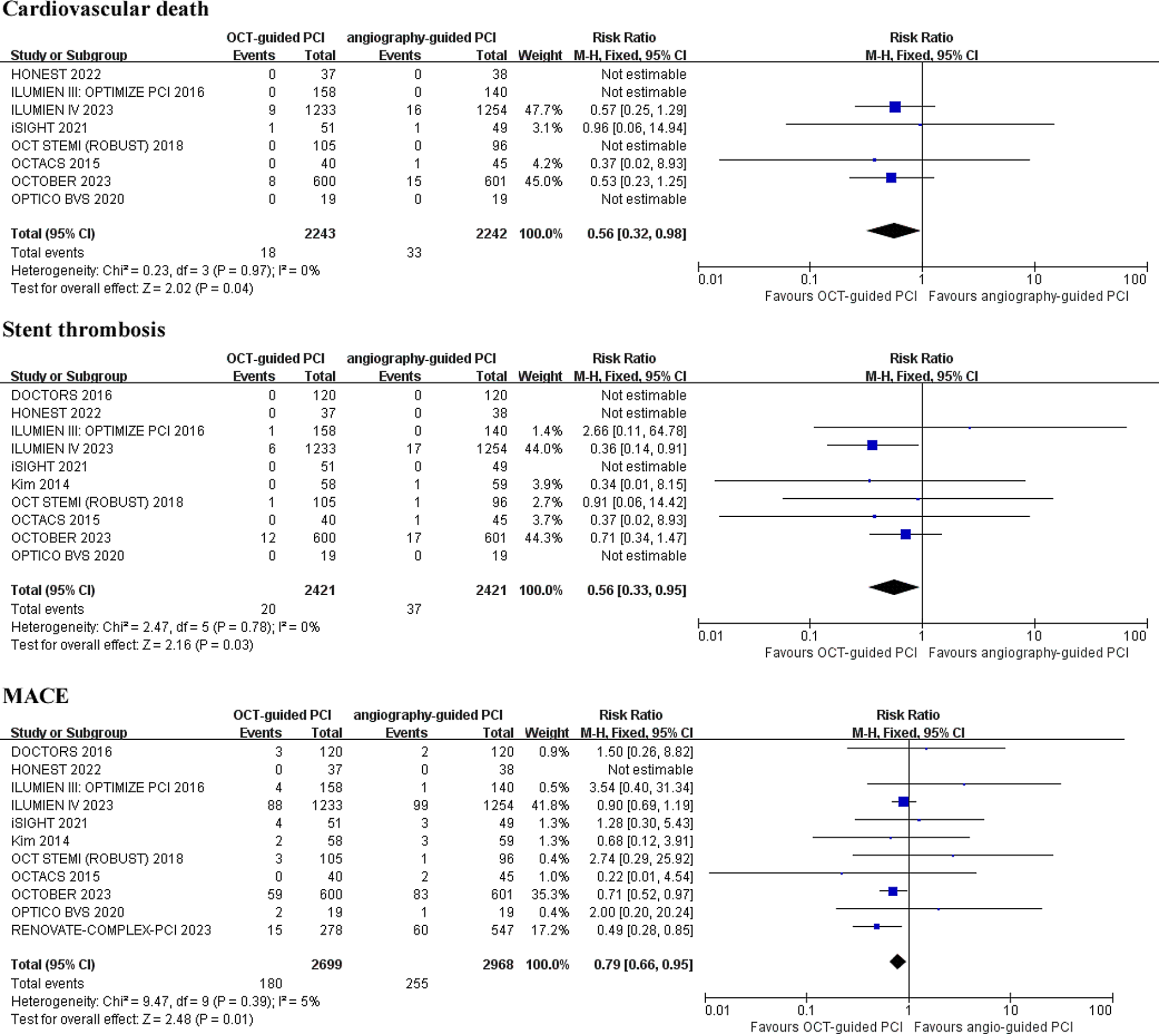
Forest plot for incidence of cardiovascular death, stent thrombosis and major adverse cardiovascular events (MACE). Risk ratio for individual studies (squares) and meta-analysis (diamonds) and 95% CI (horizontal lines) are presented

Fore cardiovascular death, an analysis of ten studies with 4,485 patients indicated that OCT-guided PCI significantly reduced the incidence of cardiovascular mortality (RR 0.56; 95% CI: 0.32–0.98; *p* = 0.04; I^2^ = 0%) (Fig. 3).

For stent thrombosis and major adverse cardiac events (MACE), ten studies with 4,842 patients and eleven studies with 5,667 patients, respectively, showed a significant reduction in both outcomes with OCT-guided PCI (RR 0.56; 95% CI: 0.33–0.95; *p* = 0.03; I^2^ = 0%) and (RR 0.79; 95% CI: 0.66–0.95; *p* = 0.01; I^2^ = 5%) (Fig. 3).

Subgroup analyses revealed that among studies exclusively involving patients with ACS, no significant difference were observed between OCT- and angiography-guided PCI regarding all-cause mortality (RR 1.06 95% CI: 0.15–7.65; *p* = 0.95; I^2^ = 0%), TLR (RR 1.91 95% CI: 0.35–10.33; *p* = 0.45; I^2^ = 0%), stent thrombosis (RR 0.60 95% CI: 0.08–4.62; *p* = 0.63; I^2^ = 0%), or MACE (RR 1.18 95% CI: 0.38–3.72; *p* = 0.77; I^2^ = 0%) as shown in Table 2.

**Table 2:** Subgroup analysis

	Number of studies	Total number of patients	OCT-guided	Angiography-guided	RR	95%CI	*p*	I^2^(%)
**ACS**								
MACE	4	601	302	299	1.18	0.38–3.72	0.77	0
All cause death	4	601	302	299	1.06	0.15–7.65	0.95	0
TLR	3	441	225	216	1.91	0.35–10.33	0.45	0
Stent thrombosis	4	601	302	299	0.60	0.08–4.62	0.63	0
**New-generation DES**								
MACE	7	4489	2245	2244	0.84	0.69–1.03	0.09	0
All cause death	6	4372	2187	2185	0.69	0.48-1.00	0.05	0
Cardiovascular death	6	4372	2187	2185	0.56	0.32–0.98	0.04	0
MI	5	4171	2082	2089	0.85	0.66–1.09	0.20	0
TLR	7	4404	2205	2199	0.96	0.71–1.30	0.78	0
Stent thrombosis	7	4489	2245	2244	0.56	0.33–0.95	0.03	0

ACS = Acute coronary syndrome; DES = Drug-eluting stents; MACE = Major adverse cardiovascular event; MI = Myocardial infarction; TLR = Target lesion revascularization

For studies utilizing new-generation DES, OCT-guided PCI significantly reduced the incidence of cardiovascular death (RR 0.56 95% CI: 0.32–0.98; *p* = 0.04; I^2^ = 0%) and stent thrombosis (RR 0.56 95% CI: 0.33–0.95; *p* = 0.03; I^2^ = 0%). Yet, the rates of all-cause mortality (RR 0.69 95% CI: 0.48-1.00; *p* = 0.05; I^2^ = 0%), MI (RR 0.85 95% CI: 0.66–1.09; *p* = 0.20; I^2^ = 0%), TLR (RR 0.96 95% CI: 0.71–1.30; *p* = 0.78; I^2^ = 0%) and MACE (RR 0.84 95% CI: 0.69–1.03; *p* = 0.09; I^2^ = 0%) showed no significant difference between the two groups.

**Fig. 4:**
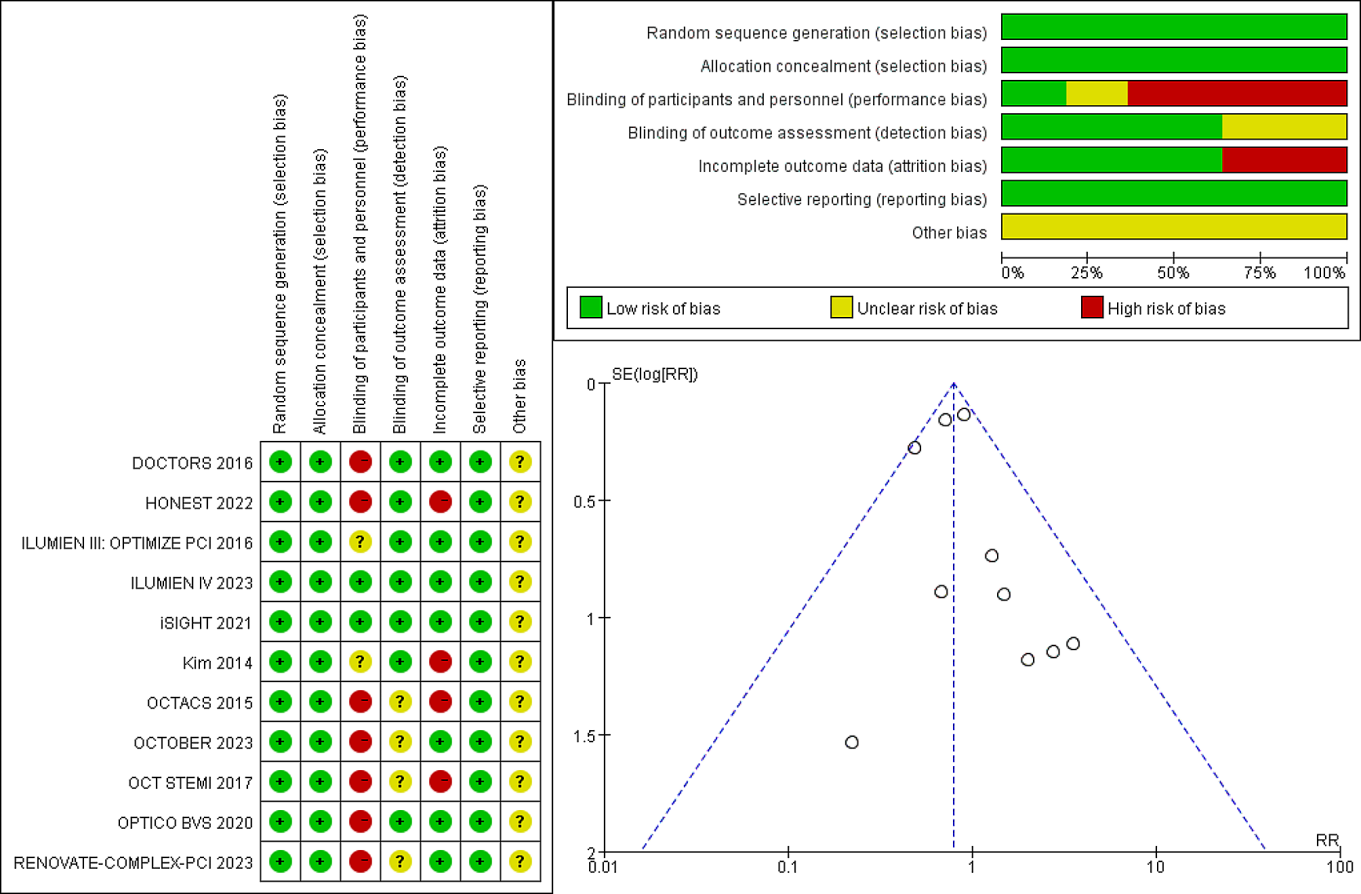
The funnel plot for incidence of major adverse cardiovascular events and risk of bias

Bias assessment indicated a low-to-moderate risk of bias across all studies. The funnel plot exhibited no asymmetry, suggesting an absence of publication bias. (Fig. 4)

## Discussion

Unlike traditional angiography that can only visualize intra-coronary lumen in two dimensions, optical coherence tomography offers an accurate and high-resolution visualization of culprit lesion morphology, enabling the optimization of stent implantation and minimization of stent-related complications. This distinctive capability has led to its widespread adoption as a crucial imaging tool in PCI. Several clinical trials have investigated OCT’s potential advantages over standard angiography-guided PCI. For example, a retrospective analysis from the Pan-London PCI registry, which included 123,764 patients treated in National Health Service hospitals in London from 2005 to 2015, demonstrated that OCT-guided PCI was associated with improved procedural outcomes, in-hospital events, and long-term survival [4]. The ILUMIEN III: OPTIMIZE PCI study found that OCT guidance was non-inferior to IVUS guidance (one-sided 97.5% lower CI − 0.70 mm [2]; *p* = 0.001), but failed to show superiority to angiography guidance (*p* = 0.12), possibly due to insufficient statistical power. Furthermore, a network meta-analysis comparing IVUS, OCT, and CA‐guided PCI, which integrated both direct and indirect evidence, found no significant differences in clinical efficacy among OCT and IVUS, or between IVUS and CA [8]. However, studies using OCT were relatively few and small, potentially lacking the power to uncover the clinical advantage of OCT guidance.

Recently, two rigorously conducted RCTs, namely “Optical Coherence Tomography Guided Coronary Stent Implantation Compared with Angiography: A Multicenter Randomized Trial in PCI " (ILUMIEN IV: OPTIMAL PCI), and “Optical Coherence Tomography Optimized Bifurcation Event Reduction” (OCTOBER), have been published, explicitly compareing OCT-guided and angiography-guided PCI [9, 10]. The OCTOBER trial demonstrated that in patients with complex coronary-artery bifurcation lesions, OCT-guided PCI was associated with a lower incidence of MACE at two years compared to angiography-guided PCI, without an increase in procedure-related complications. Meanwhile, the ILUMIEN IV trial found that OCT guidance resulted in a larger minimum stent area than angiography guidance at the index procedure, yet there was no observed difference in the incidence of target-vessel failure at two years. With such dedicated head-to-head comparative studies and a broader sample size, we have updated the pooling analysis and demonstrated the superiority of OCT-guided over angiography-guided PCI across a majority of clinical endpoints.

The role of OCT in understanding the pathology of acute coronary syndrome and guiding its intervention is being increasingly acknowledged. Pathological studies of sudden coronary death have identified plaque rupture, plaque erosion, and calcified nodules as the primary coronary plaque morphologies leading to thrombosis ACS, all of which could be clearly identified with OCT [22]. Several studies examining ACS patients with OCT have shown that plaque rupture at the culprit lesion had more pan-coronary plaque vulnerability and cause worse clinical outcomes compared to plaque erosion [23, 24]. Consequently, OCT enables a more precise assessment of the underlying pathology of ACS, offering considerable diagnostic and prognostic benefits that may potentially translate into improved clinical outcomes [25]. Moreover, in cases of coronary calcification, PCI under OCT guidance facilitates enhanced stent expansion and marked reduction in calcium thickness [26]. Despite a gradual increase in the clinical application of OCT for primary PCI in ACS, its usage rate remains substantially lower than that of IVUS [27].

Our subgroup analysis showed that OCT-guided PCI had similar clinical efficacy with angiography-guided PCI in ACS patients. This observation might be attributed to several factors. Firstly, most studies within our ACS subgroup selected angiographic endpoints, such as average coronary artery healing and the percentage of uncovered struts, leading to a limited sample size (827 patients) not powerful to detect a potential difference. On the other hand, the reported follow-up duration, not exceeding one year, is too short to demonstrate the full benefits of OCT guidance in procedural optimization.

Despite its evident advantages, the routine application of coronary intravascular imaging remains limited. A recent survey highlighted predominant reasons for reluctance to use intravascular imaging included high cost, uncertainty regarding its additional clinical benefits, and concerns over the adequacy of image interpretation skills and subsequent reaction to optimize stenting. In fact, guidance with intravascular imaging could prove more cost-efficiency over time by decreasing the need for future interventional procedures and rehospitalizations [28].

Several limitations of our study should be noted. Firstly, our analysis was unable to account for variations in clinical practice across the different studies, including the impact of various clinical condition, coronary anatomy, lesion preparations techniques, and different definitions for measured outcomes. Secondly, the protocols of PCI optimization under OCT may vary among the trials; for example, criteria for optimal stent expansion are not identical. Furthermore, the percentage of PCI procedures that satisfied the criteria for optimal stenting differed from trial to trial. Additionally, although our funnel plot did not indicate a significant publication bias among the included studies, publication bias may still exist despite our best efforts to conduct a comprehensive search.

## Conclusion

In patients undergoing PCI, the use of OCT guidance was associated with a lower incidence of cardiovascular death, stent thrombosis, and MACE when compared to angiography-guided PCI.

### Electronic supplementary material

Below is the link to the electronic supplementary material.

Supplementary Material 1

## Data Availability

The datasets used and/or analyzed during the current study are available from the corresponding author on reasonable request.

## Abbreviations

ACS: Acute coronary syndrome
CA: Coronary angiography
CAD: Coronary artery disease
CI: Confidence interval
DES: Drug-eluting stents
IVUS: Intravascular ultrasound
MACE: Major adverse cardiovascular event
MI: Myocardial infarction
OCT: Optical coherence tomography
PCI: Percutaneous coronary intervention
RCT: Randomized controlled trial
RR: Risk ratio
TLR: Target lesion revascularization
