# Supplementary material for: Optical coherence tomography (OCT) - versus angiography-guided strategy for percutaneous coronary intervention: a meta-analysis of randomized trials
Source: BMC Cardiovasc Disord. 2024 May 20;24:262. doi: 10.1186/s12872-024-03930-y (PMC11103981; doi:10.1186/s12872-024-03930-y)
Supplement: Supplementary file 1 — Supplementary Material 1 [file 12872_2024_3930_MOESM1_ESM.docx]

Supplementary Table1 Definitions of outcomes

| Trial | Definitions of outcomes |
| --- | --- |
| ILUMIEN IV | 1.Death: (Per ARC Circulation 2007; 115: 2344-2351): All deaths are considered cardiac unless an unequivocal non-cardiac cause can be established. Specifically, any unexpected death even in patients with coexisting potentially fatal non-cardiac disease (e.g. cancer, infection) should be classified as cardiac.  2.Cardiovascular death: Any death due to proximate cardiac cause (e.g. MI, low-output failure, fatal arrhythmia), unwitnessed death and death of unknown cause, all procedure related deaths including those related to concomitant treatment.  3.Myocardial infarction: According to the SCAI Definition/ Third Universal Definition  4.TLR: Repeat revascularization of the target lesion  5.Stent thrombosis: According to modified ARC definitions  6.MACE: The composite of cardiac death, myocardial infarction, and ischemia-driven target lesion revascularization. |
| OCTOBER | 1.Death: Includes death by any cause, including cardiac deaths and non‐natural causes of deaths.  2.Cardiovascular death: Encompasses death due to coronary heart disease including fatal myocardial infarction, sudden cardiac death including fatal arrhythmias and cardiac arrest without successful resuscitation, death from heart failure including cardiogenic shock, and death related the cardiac procedure within 28 days from the procedure. If death is not clearly attributable to other non‐cardiac causes it is adjudicated as cardiac death.  3.Myocardial infarction: According to the SCAI Definition/ 4^th^ Universal Definition  4.TLR: Coronary artery bypass grafting or PCI of index lesion.  5.Stent thrombosis: According to the ARC‐ criteria.  6.MACE: The composite of death from a cardiac cause, target-lesion myocardial infarction, or ischemia-driven target-lesion revascularization. |
| RENOVATE-COMPLEX-PCI | 1.Death: All death was considered cardiac death unless an unequivocal noncardiac cause can be established. Specifically, any unexpected death, even in patients with coexisting potentially fatal noncardiac disease (eg, cancer, infection), should be classified as cardiac. The cause of death (cardiac vs. non-cardiac) was adjudicated by an independent clinical events adjudication committee.  2.Cardiovascular death: Any death due to a proximate cardiac cause (eg, myocardial infarction, low output failure, fatal arrhythmia), unwitnessed death and death of unknown cause, and all procedure-related deaths, including those related to concomitant treatment, was classified as cardiac death.  3.Myocardial infarction: According to the SCAI Definition/ Third Universal Definition  4.TLR: Target lesion revascularization was defined as any repeat PCI of the target lesion or bypass surgery of the target vessel performed for restenosis or other complication of the target lesion. All target lesion revascularizations were classified prospectively as clinically indicated or not clinically indicated by the investigator prior to repeat angiography. An independent angiographic core laboratory verified that the severity of the percent diameter stenosis met the requirements for clinical indication and overruled cases where investigator reports were not in agreement. The target lesion was defined as the treated segment from 5 mm proximal to the stent and to 5 mm distal to the stent.  5.Stent thrombosis: According to the ARC‐ criteria.  6.MACE: The composite of death from cardiac causes, target-vessel myocardial infarction, or clinically driven target-vessel revascularization. |
| HONEST | 1.Death: NA  2.Cardiovascular death: NA  3.Myocardial infarction: NA  4.Stent thrombosis: NA  5.MACE: NA |
| iSIGHT | 1.Death: NA  2.Cardiovascular death: NA  3.Myocardial infarction: According to the SCAI Definition  4.TLR: Repeat revascularization of the target lesion  5.Stent thrombosis: According to the ARC‐ criteria.  6.MACE: The composite of cardiac death, nonfatal MI, and target lesion revascularization. |
| OPTICO BVS | Death: NA  Cardiovascular death: NA  Myocardial infarction: NA  TLR: NA  Stent thrombosis: NA  MACE: NA |
| OCT STEMI (ROBUST) | Death: NA  Cardiovascular death: NA  Myocardial infarction: NA  TLR: Revascularization within 5 mm to the stent edges (in-segment) on angiography.  Stent thrombosis: NA  MACE: The composite of death, MI, stent thrombosis, or repeat revascularization. |
| DOCTORS | Death: NA  Myocardial infarction: Third Universal Definition  TLR: Repeat revascularization of the target lesion.  Stent thrombosis: According to the ARC‐ criteria.  MACE: The composite of death, MI, stent thrombosis, or repeat revascularization. |
| ILUMIEN III: OPTIMIZE PCI | Death: NA  Cardiovascular death: NA  Myocardial infarction: According to the SCAI Definition  TLR: NA  Stent thrombosis: According to the ARC‐ criteria.  MACE: The composite of death, MI, stent thrombosis, or repeat revascularization. |
| OCTACS | Death: NA  Cardiovascular death: NA  Myocardial infarction: NA  Stent thrombosis: NA  MACE:NA |
| Kim | TLR:NA  Stent thrombosis: According to the ARC‐ criteria.  MACE: The composite of cardiac death, nonfatal myocardial infarction, or patients requiring target lesion revascularization. |
